# Establishing a genetic link between *FTO* and *VDR* gene polymorphisms and obesity in the Emirati population

**DOI:** 10.1186/s12881-018-0522-z

**Published:** 2018-01-17

**Authors:** Saad Mahmud Khan, Sarah El Hajj Chehadeh, Mehera Abdulrahman, Wael Osman, Habiba Al Safar

**Affiliations:** 1 College of Medical and Dental Sciences, University of Birmingham, Birmingham, UK; 2 Khalifa University Centers of Biotechnology, Khalifa University of Science and Technology, Abu Dhabi, United Arab Emirates; 3 Department of Medical Educations, Dubai Health Authority, Dubai, United Arab Emirates; 4 Biomedical Engineering Department, Khalifa University of Science and Technology, Abu Dhabi, United Arab Emirates

**Keywords:** Obesity, United Arab Emirates, *FTO* gene, Physical activity, BMI

## Abstract

**Background:**

Obesity is a metabolic disease that is widely prevalent with approximately 600 million people classified as obese worldwide. Its etiology is multifactorial and involves a complex interplay between genes and the environment. Over the past few decades, obesity rates among the Emirati population have been increasing. The aim of this study was to investigate the association of candidate gene single nucleotide polymorphisms (SNPs), namely *FTO* (rs9939609) and *VDR* (rs1544410), with obesity in the UAE population.

**Methods:**

This is a case-control study in which genomic DNA was extracted from saliva samples of 201 obese, 115 overweight, and 98 normal subjects in the United Arab Emirates (UAE). Genotyping for the variants was performed using TaqMan assay.

**Results:**

The mean Body Mass Index (BMI) ± SD for the obese, overweight, and normal subjects was 35.76 ± 4.54, 27.53 ± 1.45, and 22.69 ± 1.84 kg/m^2^, respectively. Increasing BMI values were associated with increase in values of HbA1c, systolic and diastolic blood pressure. There was a significant association observed between the *FTO* SNP rs9939609 and BMI (*p* = 0.028), with the minor allele A having a clear additive effect on BMI values. There was no significant association detected between BMI and rs1544410 of *VDR*. Moreover, significant interaction between the *FTO* rs9939609 and physical activity reduced the “AA” genotype effect on increase in BMI (*p* = 0.027).

**Conclusions:**

Our study findings indicate that the minor allele A of the rs9939609 has a significant association with increasing BMI values. Moreover, our findings support the fact that increasing BMI is associated with increasing risks of other comorbidities such as higher blood pressure, poorer glycemic control, and higher triglycerides. In addition, physical activity was found to attenuate the effect of the “AA” genotype on the predisposition to higher BMI values.

## Background

Obesity is a global public health problem with an overall prevalence of approximately 600 million [1]*.* It is described as having a body mass index (BMI) exceeding 30 kg/m^2^ [2]. Obesity has been shown to be a risk factor for many chronic diseases including diabetes, gallstones, hypertension, non-alcoholic fatty liver disease, metabolic syndrome, and cardiovascular disorders [3–5]. The disease etiology is multifactorial, involving a complex interplay between genes and the environment. Over the past few decades, obesity rates among the Emirati population have been increasing, which can be partly attributed to the lifestyle changes and urbanization evident in the country [6, 7]*.* A national survey undertaken in the United Arab Emirates (UAE) between 2009 and 2010 reported the estimated prevalence of overweight and obesity to be 65% in adult women, 28% in male adolescents and 40% in female adolescents [6]. On average, approximately 60% of Emiratis are obese or overweight making this one of the biggest health threats in the country [8].

The 12th human obesity gene map report has published 127 candidate genes that show strong associations with adiposity, fat distribution, and BMI [9]*.* Two of these genes are the Vitamin D receptor (*VDR*) gene and the fat mass and obesity associated (*FTO*) gene. The steroid hormone 1,25 Vitamin D3 plays a key role in bone homeostasis, adiposity and insulin regulation through its binding to the Vitamin D receptor (VDR), a nuclear transcription factor, which then interacts with various cell signaling pathways [10]. In mouse models on a high fat diet, Vitamin D supplementation increased fat oxidation limiting the weight gained [11]. However, the effects of Vitamin D remain unclear with some studies reporting no effects of Vitamin D supplementation on body fat, weight, visceral/subcutaneous adipose tissue and inflammation [12]. On the contrary, a study in transgenic mice showed that over-expression of human *VDR* in adipocytes caused decreased energy expenditure and induced obesity [13]. The rs1544410 *VDR* SNP is one of the most well studied *VDR* SNPs in the Arab population with mixed results reported for its association with obesity traits especially within the Gulf Cooperation Council countries [14, 15]. A Saudi study showed that individuals with the “GTA” haplotype for the Taq1 (rs731236), Bsm1 (rs1544410), and Apa1 (rs7975232) SNPs respectively had upregulated inflammatory genes expression and increased plasma lipopolysaccharide levels, which may partly explain how these SNP alleles predispose to higher BMI values and obesity development [16]. Another study reported that the “TT” risk genotype for the Bsm1 variant was shown to be associated with an increased risk of Vitamin D deficiency [17]. Along with the obesity epidemic, Vitamin D deficiency is heavily prevalent in the UAE with estimates ranging approximately between 85% and 97% [18, 19]. Interestingly, obese subjects have been shown to have high prevalence of low Vitamin D levels compared to healthy individuals [20]. Hence, we were interested in investigating if any association existed between the rs1544410 SNP and obesity.

The *FTO* gene, particularly the rs9939609 variant, is the most well studied gene globally that has been implicated for its role in obesity development. Frayling et al. was the first to identify the rs9939609 on intron 1 as an obesity risk SNP, which was found to be in high linkage disequilibrium (LD) (r^2^ > 0.5) with a region spanning 47 kb including intron 1, 2 and exon 2 of the *FTO* gene [21]. Transcribed *FTO* has shown to be highly expressed in the hypothalamus region of the brain, controlling appetite and energy expenditure [22]. A European study discovered that those who possessed the rs9939609 “AA” risk genotype had decreased hunger reduction and suppression of acyl-ghrelin post meals along with altered neural responses to food cues [23]. Consequently, some *FTO* polymorphisms, especially the rs9939609 polymorphism, have been shown to have a strong association with BMI, adiposity and waist circumference [24]. Nevertheless, variations in association of these gene SNPs with susceptibility to obesity have been shown in different populations. While a link between *FTO* gene variants and higher percentage of fat was observed in some populations such as Danish adults and Scottish children, this association was not significant in the Chinese Han population, indicating a probability of ethnic-specific associations [25–27]*.*

Due to the aforementioned reasons and since no study to date has analyzed the correlation between these two key genes and obesity in Emiratis, the aim of this study was to investigate the association between *FTO* (rs9939609) and *VDR* (rs1544410) polymorphisms with obesity in the UAE population.

## Methods

### Subjects and sample collection

This case control study included four hundred and fourteen participants (*n* = 414), which consisted of 237 female subjects and 177 male subjects. The participants were enrolled during their routine visit to endocrinology clinics in Abu Dhabi, United Arab Emirates between the period of June 2014 and May 2015. Several biochemical tests and clinical assessments along with a lifestyle questionnaire were completed at the clinic for each participant. These included weight and height assessment, which were used to calculate the Body Mass Index for each participant. The study inclusion criteria were age ≥ 25, UAE national, non-pregnant women and having the ability to give consent.

Individuals were classified as normal weight (98 healthy controls with no chronic diseases), overweight (115 subjects), and obese (201 subjects) according to the World Health Organization (WHO) BMI classification criteria [28]. The age of the participants ranged from 29 to 95 years, where the mean age of healthy subjects, overweight subjects and obese subjects was 22.69 ± 1.84, 27.54 ± 1.45 and 35.76 ± 4.54 respectively. The percentage of healthy, overweight and obese male subjects was 34.69%, 56.52% and 38.81% respectively. A total of 263 individuals among those who were overweight and obese had Type 2 Diabetes. We had information about medication taken for 30.4% of the study subjects, however, we did not include this in any analysis to avoid bias and misleading results.

### DNA extraction and quantification

One milliliter of saliva was collected from each study subject using the Oragene OGR-500 kit (DNA Genotek, Ottwa, Canada). The prepIT®L2P system (DNA Genotek, Ottawa, Canada) was used to extract genomic DNA from the collected saliva samples. Extracted DNA was quantified using the Nanodrop 2000C Spectrophotometer (Thermo Scientific, Wilmington, USA).

### Genotyping

*FTO* (rs9939609) and *VDR* (rs1544410) variants were genotyped using TaqMan assay (Applied Biosystems, Foster City, USA), where fluorescently labeled (FAM™ and VIC®) minor groove binder (MGB™) probes (Applied Biosystems, Foster City, USA) were used. The *VDR* polymorphism rs1544410 sequence of Primers (F: 5’-TTCCTGGGGCCACAGAC-3′; R: 5’-GAGCAGAGCCTGAGTAT-3′) and MGB™ probes (Probe 1: VIC-GCCTGC[A]CATTCCC; Probe 2: FAM-GCCTGC[G]CATTCCC) and the *FTO* polymorphism rs9939609 sequence of Primers (F: 5’-GGTTCCTTGCGACTGCT-3′; R: 5’-AACAGAGACTATCCAAG-3′) and MGB™ probes (Probe 1: VIC-GAATTT[A]GTGATGC; Probe 2: FAM-GAATTT[T]GTGATGC) were used to identify the respective polymorphisms. The quantitative PCR was carried out using the MicroAmp® Fast Optical 96-Well Reaction Plate. Each well contained 10 ng of DNA, 5 μl of GTXpress Master Mix (Applied Biosystems, Foster City, USA) and 0.5 μl of Taqman real-time PCR assays (20X diluted), amounting the total volume to 10 μl. Each plate had a dedicated control well to ensure reliability of the results. Amplification reactions began with incubation at 95 °C for 20s, followed by 40 cycles of 95 °C for 3 s (denaturing) and 60 °C for 20s (annealing/extension). DNA amplification was performed in the ViiA™ 7 Real-time PCR system (Applied Biosystems, Foster City, USA) with the incorporated software for single nucleotide polymorphisms (SNPs) genotyping (Applied Biosystems, Foster City, USA) used to identify the alleles present.

### Statistical analyses

All statistical tests performed in this study were two-tailed and *p*-values ˂ 0.05 were considered to be statistically significant. Statistical analyses were performed using R version 3.3.1 (R Foundation, Vienna, Austria) and STATA version 13 (STATA Corp., TX, USA). All continuous variables were expressed as mean ± SD. The genotype frequencies were tested for Hardy–Weinberg equilibrium using a chi-square test through the https://ihg.gsf.de/ihg/index_engl.html website. Fisher exact confidence intervals were drawn for relative risk estimates and calculated with a 95% confidence interval. The relationships between the three subject groups (obese; overweight; and normal) and the demographic data plus biochemical tests of the participants were analyzed using the analysis of variance (one-way ANOVA) followed by post hoc statistics, respectively. The univariate correlation analyses were produced using the correlation test with a Fisher’s transformation.

Normal distribution of BMI in the study was ensured by using *Z* scores of the BMI values to find associations of BMI with clinical/biochemical characteristics and SNPs. The multiple linear regression analysis was used to confirm the association between BMI (corrected as *Z* scores to ensure a standard normal distribution) and the different SNPs after excluding confounders. The “SNPassoc” package of the R version 3.3.1 (R Foundation, Vienna, Austria) was used to investigate any gender-specific associations between our SNPs and BMI using the log-additive model.

The “SNPassoc” package of the R version 3.3.1 (R Foundation, Vienna, Austria) was also used to perform gene-gene interaction analysis. This was followed by looking at the effect of *FTO* and *VDR* SNPs genotype combinations on BMI values using ANOVA, Tukey post hoc test and “gplots” package of the R version 3.3.1 (R Foundation, Vienna, Austria). Lastly, we performed interaction analysis between our *FTO* SNP rs9939609 and physical activity looking at the effect on risk of increased BMI.

## Results

The demographics for the study subjects along with their clinical and biochemical characteristics are detailed in Table 1. There was a significant difference observed in mean age, BMI, systolic blood pressure and HbA1c levels among obese, overweight and normal weight subjects (*p* = 0.0005, *p* < 0.00001, *p* = 0.0002 and *p* = 0.034 respectively), with a clear linear increase towards increase in body weight. Furthermore, triglyceride levels were highest in the obese individuals (1.79 ± 2.23), followed by the overweight individuals (1.58 ± 1.56) and the lowest in the normal weight individuals (1.16 ± 0.58), however, these differences were not statistically significant (Table 1).

**Table 1:** Clinical and biochemical characteristics of the study subjects

Category	Variable	Obese (*n* = 201)	Overweight (*n* = 115)	Normal (*n* = 98)	*p-*value
Demographic data	Age (years)	57.48 ± 11.80	57.66 ± 12.05	52.10 ± 15.56	0.001*
	BMI (kg/m^2^)	35.77 ± 4.55	27.56 ± 1.44	22.72 ± 1.84	< 0.00001*
	Systolic blood pressure (mmHg)	131.05 ± 16.18	126.63 ± 16.70	122.68 ± 19.28	0.0006*
	Diastolic blood pressure (mmHg)	74.30 ± 10.71	72.74 ± 11.87	71.58 ± 11.94	0.155
Biochemical tests	HbA1c (%)	7.24 ± 1.48	7.19 ± 1.48	6.57 ± 1.17	0.033*
	Triglyceride (mmol/L)	1.80 ± 2.25	1.59 ± 1.56	1.15 ± 0.58	0.091
	Total Cholesterol (mmol/L)	4.20 ± 1.45	4.19 ± 1.68	4.37 ± 1.11	0.738
	HDL Cholesterol (mmol/L)	1.30 ± 0.68	1.17 ± 0.40	1.30 ± 0.38	0.162
	LDL Cholesterol (mmol/L)	2.23 ± 1.03	2.36 ± 1.29	2.54 ± 1.02	0.199
Lifestyle data	Smoker (Yes)	8.08%	7.96%	10.41%	0.769
	Physically active (Yes)	53.77%	57.39%	43.75%	0.124

All continuous variables are presented as mean ± standard deviation. All binary variables are presented as percentages Abbreviations- *BMI* Body mass index, *HbA1c* Glycosylated hemoglobin, *HDL* High density lipoprotein, *LDL* Low density lipoprotein, *n* Number of individuals. *Significant *p-*values< 0.05 are in bold

To determine the factors that may affect development of obesity, a part of the SNPs, we tested the associations of *Z* scores of the BMI values with the clinical and laboratory parameters of the research subjects. The results showed a significant correlation between BMI and age (*p* = 0.002), and BMI with systolic blood pressure (*p* = 0.007) as shown in Table 2**.** Therefore, we used these two parameters for adjustment and further analyses.

**Table 2:** Association between BMI and clinical and laboratory variables of the study subjects

Clinical or biochemical variant	Estimate	SE	*P*
Age	−0.017	0.005	**0.002**
Systolic blood pressure	0.011	0.004	**0.007**
Diastolic blood pressure	−0.001	0.006	0.800
HbA1c	0.056	0.038	0.150
Triglyceride	−0.027	0.060	0.650
Total Cholesterol	0.174	0.169	0.300
HDL Cholesterol	0.032	0.115	0.780
LDL Cholesterol	−0.245	0.181	0.180
Vitamin D	0.0004	0.002	0.850

Abbreviations- *HbA1c* Glycosylated hemoglobin, *HDL* High density lipoprotein, *LDL* Low density lipoprotein, *P p* value, *SE* Standard error. *Significant *p* values< 0.05 are in bold

### Whole cohort SNPs-BMI association analyses

**Fig. 1:**
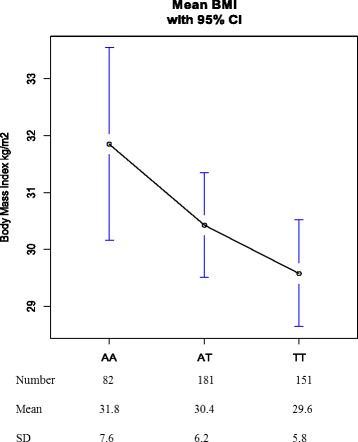
Association between BMI and genotypes of *FTO* SNP (rs9939609)

The association of *Z* scores of the BMI values with genotypes of *FTO* and *VDR* is shown in Table 3. For the *FTO* SNP rs9939609, there was a significant difference observed between the SNP genotypes and BMI (*P*_*unadjusted*_ = 0.011), with a clear additive effect of allele A of the SNP (Fig. 1). This association remained significant following adjustment for age and systolic blood pressure values (*P*_*adjusted*_ = 0.013). No significant difference was observed for the *VDR* SNP rs1544410 with BMI (*P*_*adjusted*_ = 0.21).

**Table 3:** Association between BMI and SNPs selected for the study

SNPs	Gene	Alleles (1 / 2)	MAF	Estimate	SE	P*
rs9939609	*FTO*	A / T	0.416	−0.166	0.066	**0.013**
rs1544410	*VDR*	C / T	0.486	−0.082	0.066	0.210

Abbreviations- *BMI* Body mass index, *FTO* Fat mass and obesity-associated protein*, VDR* Vitamin D receptor, *SNP* Single nucleotide polymorphism*,* Allele 1 / 2: minor / major alleles. Genotypes with significant *p* < 0.05 are marked in bold. *P values are adjusted for age of subjects and their systolic blood pressure values

### Gender specific SNPs-BMI association analyses

The association of the SNPs rs99396609 and rs1544410 with BMI in each gender are reported in Table 4. After gender stratification, our association analysis revealed that there was a significant association for females between BMI and the rs9939609 SNP (*p* = 0.048) with the “AA” genotype predisposing to increased BMI, however, no significance was observed for the rs1544410 SNP (*p* = 0.796). There was no significant association observed for males between BMI and both our SNPs, rs9939609 (*p* = 0.112) and rs1544410 (*p* = 0.165).

**Table 4:** Association of rs9939609 (FTO) and rs1544410 (VDR) with BMI in males and females

Log-additive model		*FTO* (rs9939609)			*VDR* (rs1544410)	
Gender	Genotype	Mean BMI (kg/m^2^)	*p*-value	Genotype	Mean BMI (kg/m^2^)	*p-*value
Male	*AA*	31.67	0.112	*CC*	32.30	0.165
	*AT*	29.77		*CT*	28.63	
	*TT*	29.44		*TT*	30.41	
Female	*AA*	31.97	0.048*	*CC*	30.67	0.796
	*AT*	30.99		*CT*	30.91	
	*TT*	29.67		*TT*	30.38	

*significant p-values shown in bold (*p* < 0.05). Abbreviations- *BMI* Body mass index, *FTO* Fat mass and obesity-associated protein, *VDR* Vitamin D receptor, *SNP* single nucleotide polymorphism

**Table 5:** Interaction of the SNP rs9939609 (FTO) with physical activity (Yes/No) effect on BMI

Gene (SNP)	Physical Activity	Genotypes	Frequency	Mean BMI (kg/m^2^)	*p-interaction*
*FTO (*rs9939609)	Yes	*AA*	33	31	0.027*
		*AT*	78	*31*	
		*TT*	74	*30*	
	No	*AA*	44	34	
		*AT*	96	*30*	
		*TT*	69	*30*	

*significant *p*-values adjusted for age and systolic blood pressure (*p* < 0.05). Abbreviations- *BMI* Body Mass Index**,** *FTO* Fat mass and obesity-associated protein, *SNP* Single nucleotide polymorphism

### *FTO*-*VDR* interaction analysis

**Fig. 2:**
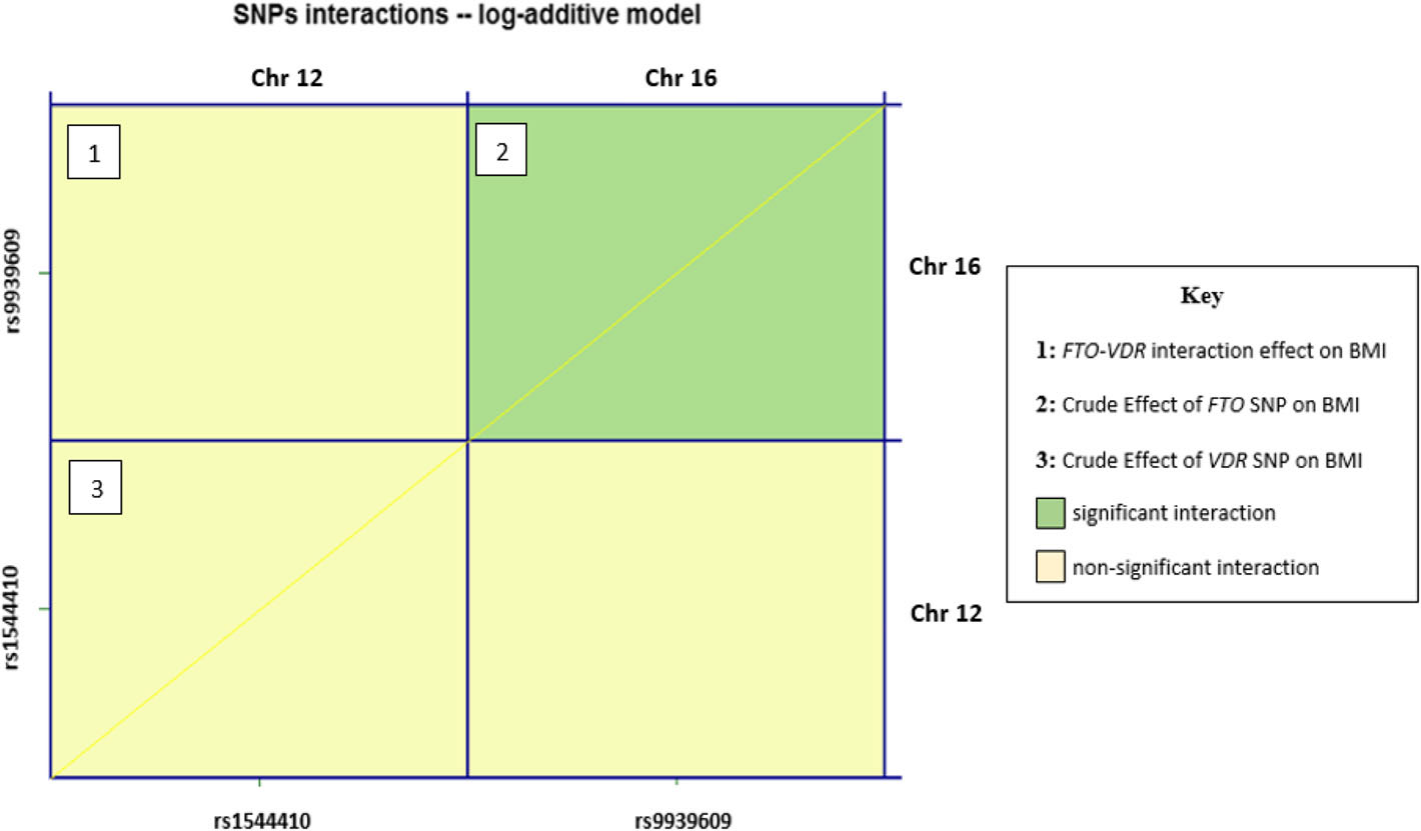
Gene-Gene interaction of *FTO* rs9939609 with *VDR* rs1544410 on BMI values

To test the effects of the two SNPs together, we performed an interaction analyses as shown in Fig. 2 using the log-additive model. There appears to be no epistasis between the *FTO* rs9939609 SNP located on Chromosome 16 position 53,786,615 and the *VDR* rs1544410 SNP located on Chromosome 12 position 47,846,052 for BMI values (*p-interaction* = 0.213). Only the crude effect of *FTO* rs9939609 SNP is significant for BMI values (*p* = 0.011).

**Fig. 3:**
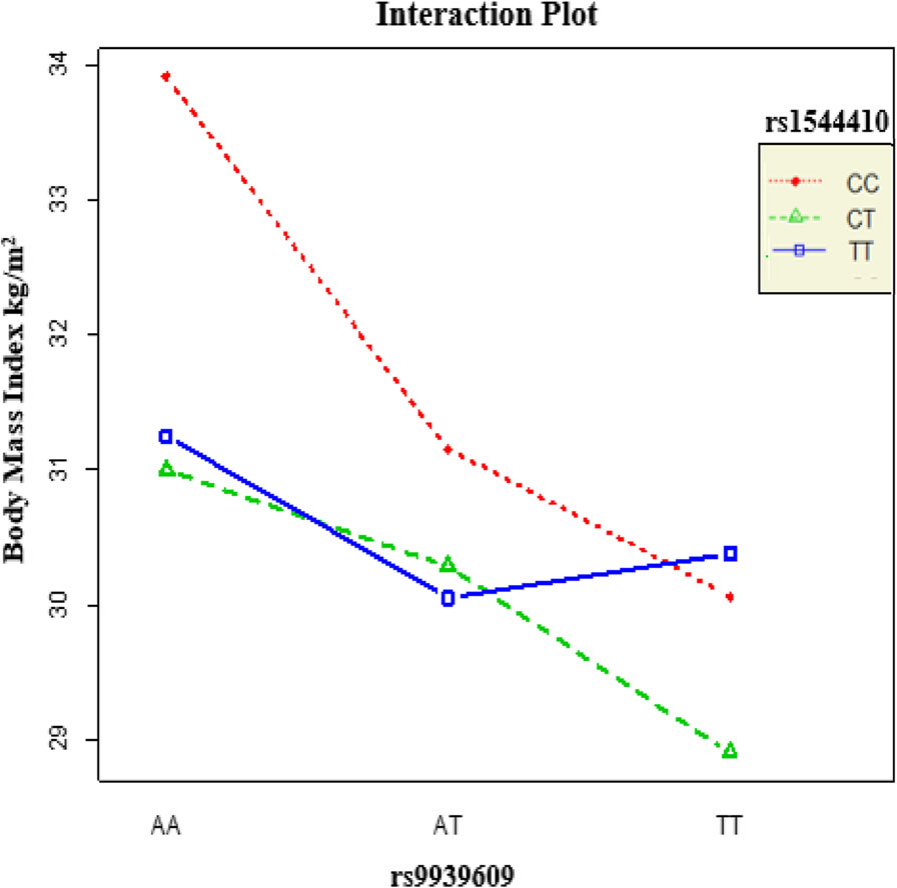
Body mass index values for genotype combinations of SNPs rs9939609 and rs1544410

Despite no evident epistasis, we went on to analyze which gene combinations confer the highest increased BMI risk that is illustrated in Fig. 3. The *FTO* “AA” genotype and *VDR* “CC” genotype combination gave the highest BMI (higher than the mean BMI for *FTO* “AA” genotype alone) whereas the lowest BMI value obtained was for the *FTO* “TT” genotype and *VDR* “CT” combination. A significant difference was found between these two combinations (AA:CC - TT:CT; *p* = 0.016).

### *FTO*-physical activity interaction and increased BMI risk

We also conducted an interaction analysis (Table 5) looking at the effect of physical activity on the *FTO* SNP rs9939609 genetic risk for increased BMI values. There was a significant interaction between physical activity and rs9939609 for its effect on the risk of increased BMI after adjustment with age and systolic blood pressure (*p-interaction* = 0.027) as shown in Table 5. Our results showed that being physically active reduces the effect of the rs9939609 “AA” genotype towards increased BMI risk, with those who were physically active having a lower BMI value (mean = 31 kg/m^2^) than those who were not active (mean = 34 kg/m^2^) with the “AA” mutant genotype.

## Discussion

Obesity is a known prevalent disorder among the Emirati population. Due to poor representation of Arab countries in the Hap Map and 1000 Genome projects, researchers in the Emirates have been conducting genome wide association studies (GWAS) in an attempt to identify associations between gene SNPs and chronic diseases [29].

In this study, we investigated the genetic link between the SNPs rs9939609 of *FTO* and rs1544410 of *VDR* genes with the obesity phenotype in the Emirati population for the first time. Our results show that there was a significant association between the *FTO* rs9939609 genotype and obesity, where the homozygous mutant AA genotype predisposed individuals to increased BMI. In addition, after excluding the effects of age, systolic blood pressure, diastolic blood pressure, and triglyceride levels, BMI decreases with the presence of the wild type ‘T’ allele (estimate = − 0.144 for A per T allele). Furthermore, carriers of the AA genotype had a 2.2 point increase in their mean BMI in comparison to those who were carriers of the TT genotype (Fig. 1), indicating a strong contribution of *FTO* on obesity susceptibility. This is in concordance with many other studies conducted in the Asian and European populations that strongly linked the *FTO* rs9939609 SNP to obesity susceptibility, with the ‘T’ allele indicating a protective role [30–33]. A meta-analysis of 12 studies including 5000 obese and 9853 controls showed a strong association of the *FTO* rs9939609 polymorphism with obesity among children and adolescents from several Caucasian populations [34]. Moreover, a multicenter trial in Europe showed strong association with severe obesity in bariatric patients (*p* =  9.2 × 10^− 8^, OR = 1.47) as well [35]. The association of the risk allele “A” with higher leptin to fat mass ratio, higher levels of thyrotropin and lower resting energy expenditure is a likely explanation for its predisposition to increased obesity risk [36]. However, there have been studies in African American and Chinese Han population that show no association of the “A” allele with higher BMI risk [27, 37]. This lack of association can be explained by weaker linkage disequilibrium patterns that exist between the *FTO* variants in African Americans and the Chinese Han population as compared to the European population [38]. Moreover, in the Chinese Han population, the lower allele “A” frequency as compared to the European population may be a potential contributing factor to this lack of association observed (HCB = 0.116; CEU = 0.460) [39]. Studies on the Greek and Spanish Roma cohorts have also reported non-association of obesity with this SNP [40, 41].

The 1,25-dihydroxyvitamin D3 steroid and the Vitamin D Receptor have been shown to inhibit adipogenesis in the 3 T3-L1 preadipocyte cell line [42]. However, the role of Vitamin D in obesity development still needs further research due to conflicting study findings. Vimaleswaran et al. showed that no association of Vitamin D pathway genes, including *VDR*, existed with waist circumference, BMI and other obesity related traits [43]. Moreover, it remains unclear whether it is Vitamin D deficiency and *VDR* that contributes to obesity or is it obesity that causes low Vitamin D levels as adipose tissue has been shown to sequester Vitamin D leading to reduced plasma levels [10]. Our results demonstrated that the SNP rs1544410 of the *VDR* polymorphism was not associated with BMI in Emiratis (*p* = 0.31), this being consistent with study findings from the Bahraini population which demonstrated the *VDR* gene SNPs rs731236 and rs12721377 were not related to BMI (rs1544410 tested in this study and rs731236 tested in the Bahraini population are in complete linkage disequilibrium; D` = 1, r^2^ = 1) [15]. On the other hand, reports showed that T carriers of rs1544410 tend to have lower BMI values [44]. Furthermore, the allele ‘C’ and the dominant homozygous genotype CC in rs1544410 were reported to be associated with metabolic syndrome, while the allele T and the heterozygous genotype CT seemed to play a protective role [45]. Other studies that had contrasting findings to ours was the study by Al-Daghri et al. that showed the *VDR* SNPs rs1544410 “T” allele increased the risk of obesity in Saudis (*p* = 0.028) [16]. Similar findings were replicated by a study on the Qatari cohort, where the “T” allele was associated with adiposity measures including increased BMI (*p* = 0.009) and body fat percentage (*p* = 0.04) [14].

We also performed association analysis for both our SNPs, rs9939609 and rs1544410, with BMI after gender stratification. Frayling et al. reported the association of BMI with rs9939609 for both genders [21]. Interestingly, in our study the only association found to be significant was in females between *FTO* rs9939609 and obesity. These findings were in line with a study in Swedish children that reported an association in girls for the rs9939609 SNP with BMI (*p* = 0.004) and obesity (*p* = 0.006), which was not significant for boys [46].

Despite no evidence of any epistasis, we then went on to perform genotype combination analysis for rs9939609 and rs1544410, which showed that the *FTO* “AA” and *VDR* “CC” genotype combination and the *FTO* “TT” and *VDR* “CT” combination gave the highest and lowest mean BMI respectively (AA:CC-TT:CT; *p* = 0.016). This supports the fact that obesity is polygenic phenotype where multiple gene variations are expected to be involved. Our result also suggests that even though *VDR* (rs1544410 SNP) on its own did not show any genetic association with obesity in this cohort, but when combined with *FTO* (rs9939609) they contribute to the increased BMI values. However, we cannot compare our findings to other Arabian Gulf countries as no such study has been conducted to identify any link, in particular the *FTO* rs9939609 gene variant, with BMI. Consequently, this study warrants for other regional countries to carry out genome-wide analyses in an attempt to discover key genes that predispose particular populations to the obesity phenotype. Interestingly, it has been shown that leading an active physical lifestyle, a common problem in Arabian Gulf region, can reduce the impact of the AA genotype of rs9939609 in *FTO* gene towards adiposity measures including increased BMI and waist circumference in European adults [47–49]. Our study results were in line with these findings where a significant interaction was observed between physical activity and rs9939609 (*p-interaction* = 0.027) as shown in (Table 5) on BMI values. Those with the “AA” risk genotype who were physically active had a lower mean BMI than those who were not physically active. However, a study in African American and European youth showed no such SNP-physical activity interaction [50].

This was the first study of its kind to investigate the association of these two candidate genes with obesity in the UAE. One limitation of our study is the small sample size chosen that limited statistical power. To strengthen our findings, further replication and fine mapping in a larger cohort of Arab population samples will be essential to validate the results presented here.

## Conclusions

In conclusion, the *FTO* SNP rs9939609 “AA” genotype is associated with higher BMI values. Moreover, physical activity was found to attenuate the effect of this mutant allele on risk of higher BMI. Subsequently, we propose those Emiratis that might be predisposed to increased BMI (due to positive family history) will benefit from screening, and consequently can take extra lifestyle measures to prevent obesity onset in the already obesity threatened region.

## Abbreviations

BMI: Body mass index
*FTO*: Fat mass and obesity associated (*FTO*) gene
UAE: United Arab Emirates
*VDR*: Vitamin D receptor (*VDR*) gene
